# Assessment of trace and macroelement accumulation in cyprinid juveniles as bioindicators of aquatic pollution: effects of diets and habitat preferences

**DOI:** 10.1038/s41598-024-61986-4

**Published:** 2024-05-17

**Authors:** Krisztián Nyeste, Nurfatin Zulkipli, Ifeanyi Emmanuel Uzochukwu, Dóra Somogyi, László Nagy, István Czeglédi, Sándor Harangi, Edina Baranyai, Edina Simon, Sándor Alex Nagy, Iliana Velcheva, Vesela Yancheva, László Antal

**Affiliations:** 1 Department of Hydrobiology, University of Debrecen, P.O. Box 57, Debrecen, 4032 Hungary; 2 National Laboratory for Water Science and Water Safety, University of Debrecen, Debrecen, 4032 Hungary; 3 Pál Juhász-Nagy Doctoral School of Biology and Environmental Sciences, University of Debrecen, Debrecen, 4032 Hungary; 4 HUN-REN Balaton Limnological Research Institute, Tihany, 8237 Hungary; 5 National Laboratory for Water Science and Water Security, HUN-REN Balaton Limnological Research Institute, Tihany, 8237 Hungary; 6 Department of Inorganic and Analytical Chemistry, Agilent Atomic Spectroscopy Partner Laboratory, University of Debrecen, Debrecen, 4032 Hungary; 7 Department of Ecology, University of Debrecen, Debrecen, Debrecen, 4032 Hungary; 8 ELKH-DE Anthropocene Ecology Research Group, Debrecen, 4032 Hungary; 9 Department of Ecology and Environmental Conservation, Faculty of Biology, Plovdiv University, 4000 Plovdiv, Bulgaria

**Keywords:** Heavy metals, Fish, Habitat preference, Trophic level, Diet, Pollution, Ichthyology, Environmental sciences, Metals

## Abstract

Juveniles of three cyprinids with various diets and habitat preferences were collected from the Szamos River (Hungary) during a period of pollution in November 2013: the herbivorous, benthic nase (*Chondrostoma nasus*), the benthivorous, benthic barbel (*Barbus barbus*), and the omnivorous, pelagic chub (*Squalius cephalus*). Our study aimed to assess the accumulation of these elements across species with varying diets and habitat preferences, as well as their potential role in biomonitoring efforts. The Ca, K, Mg, Na, Cd, Cr, Cu, Fe, Mn, Pb, Sr, and Zn concentration was analyzed in muscle, gills, and liver using MP-AES. The muscle and gill concentrations of Cr, Cu, Fe, and Zn increased with trophic level. At the same time, several differences were found among the trace element patterns related to habitat preferences. The trace elements, including Cd, Pb, and Zn, which exceeded threshold concentrations in the water, exhibited higher accumulations mainly in the muscle and gills of the pelagic chub. Furthermore, the elevated concentrations of trace elements in sediments (Cr, Cu, Mn) demonstrated higher accumulation in the benthic nase and barbel. Our findings show habitat preference as a key factor in juvenile bioindicator capability, advocating for the simultaneous use of pelagic and benthic juveniles to assess water and sediment pollution status.

## Introduction

Trace element pollution seriously threatens the biosphere, especially aquatic ecosystems^1,2^. These pollutants and their environmental importance have been widely known since the Minamata disease, one of the first documented human diseases caused by trace element pollution^3,4^. In aquatic ecosystems, trace elements have two primary sources: the natural geological background and anthropogenic activities, such as agriculture, industry, and mining^5,6^. Pollution of the aquatic environment with trace elements can be especially harmful due to their toxicity, non-biodegradable features, and long persistence^7^. When these elements enter aquatic ecosystems, they can accumulate in the water, sediments, and aquatic organisms^8^.

The chemical analysis of water and sediments is a traditional tool for monitoring trace element pollution^9^. Due to their bioaccumulation and biomagnification susceptibility, the trace element concentrations in aquatic organisms could be relatively higher than those in water and sediments^10–12^. Among these organisms, fish have attracted considerable interest in monitoring aquatic contamination ^13–15^. Fish can provide a longer-term, complex sight of the pollution status of their environment because all environmental factors (such as trace element contamination) integrate into their organs during their lifespan^8,16^. Furthermore, fish is one of the most important food sources for humans; thus, monitoring their trace elements patterns is essential to ensure the safety of fish consumption^17–19^.

Fish can take up trace elements by several routes, e.g., through the skin and gills or the digestive system^20,21^. Therefore, trace element accumulation in fish depends on several factors, such as habitat preference and the diet of different species^22^. For example, several studies suggest that species with benthic habitat preferences and piscivorous or benthivorous diets may accumulate higher concentrations of trace elements^22–24^, while others found that pelagic and omnivorous fish accumulate more trace elements^25^. At the same time, the trace element patterns are different in the different tissues of a given species because of their various physiological features^20,26^. In general, the trace element concentrations are higher in the liver and gills compared to those in muscle tissue; hence, they have been widely used as bioindicator organs for trace element pollution of aquatic ecosystems in monitoring and risk assessment programs^27,28^. As a secondary target of bioaccumulation of trace elements, the muscle generally contains lower concentrations of trace elements than the liver or gills^29^. However, the muscle is the main part of the fish consumed by humans; therefore, monitoring its trace element pattern is equally important^19,30^.

Traditionally, most of the field studies focused on the trace element patterns of adult fish^6,31,32^. However, the bioaccumulation of trace elements is influenced not only by habitat preference and diet but also by age and size^18,29,33–38^. During the last decade, a considerable amount of research highlighted the bioindicator capability of juveniles to assess the trace element pollution of aquatic ecosystems^18,29,39^. The trace element concentrations of juveniles can be higher than those of adult fish due to various mechanisms, e.g., increased sensitivity, inadequately developed detoxification system, and higher relative metabolic rate^18,29,36,38^. In addition, juveniles integrate the environmental effects of the current year only; therefore, as a result of the phenomena mentioned above, they can be used as bioindicators of the recent trace element pollution^18^. However, there is a scarcity of data regarding the functional traits, such as diet and habitat preference, that influence the bioindicator characteristics of juveniles inhabiting a polluted aquatic ecosystem.

Nase [*Chondrostoma nasus* (Linnaeus, 1758)] is a widespread cyprinid species in Western and Central Europe, with a benthic habitat preference^40^. Its primary habitat is moderate to fast-flowing large to medium-sized rivers with rock or gravel bottoms^40^. The larvae feed mainly on zooplankton, while the juveniles and adults feed almost exclusively on benthic algae cleaned up from rock or stone surfaces using their specific lower lip with thick cornified sheath^35,40^.

Barbel [*Barbus barbus* (Linnaeus, 1758)] is one of the most frequent cyprinid species in Europe, living in medium-sized and large rivers with moderate or fast-flowing water and gravel bottom^40^. It has a benthic habitat preference and a benthivorous diet, feeding chiefly on benthic invertebrates and detritus^40^.

Chub [*Squalius cephalus* (Linnaeus, 1758) previously *Leuciscus cephalus*] is also one of the most common and widespread cyprinid species in Europe^40^. Chub is most abundant in small rivers and large streams, even in slow-flowing lowland rivers and mountain streams^40^. It has a pelagic habitat preference and omnivorous diet, feeding on various aquatic organisms^40^.

At the beginning of their developmental stages, larvae and early juveniles of these species primarily rely on zooplankton as their main food source, and they typically inhabit shallow shoreline habitats^40^. However, after they advance through early development, significant ontogenetic shifts in both feeding behavior and habitat preference become apparent^41–44^, i.e., older juveniles gradually shift towards consuming species-specific prey items, adopting similar habitat preferences to adults several months after hatching^45–47^.These three cyprinid species are popular game fish and are commercially exploited in several European countries^22,40,48^. Furthermore, they are considered good bioindicators of the health of aquatic ecosystems^22,35,49–60^.

The Szamos River is one of the most polluted rivers in Central Europe, mainly due to the intensive mining activities in Romania^11,61^. In 2000, 100,000 m^3^ of cyanide and trace element-rich liquid waste was released into the Water System of the Szamos River^62^. The pollution of the Szamos River and its recipient, the Tisza River, has been named the worst environmental disaster in Europe since the Chernobyl nuclear leak in 1986^62,63^. After this ecological catastrophe, the trace element concentrations rapidly decreased in the Szamos River^61,64,65^. Nonetheless, a significant level of pollution with Cd, Cr, Cu, Mn, Pb, Sr, and Zn was observed in 2013 based on earlier studies^11,18,66^. Subsequently, according to water chemistry data from the National Environmental Information System of Hungary, there was no notable pollution in the Szamos River.

This study aimed to investigate the trace element accumulation pattern in different tissues of nase, barbel, and chub juveniles from the polluted aquatic ecosystem of the Szamos River. Previous research highlighted the potential of juveniles as effective bioindicators of recent trace element pollution^18,29,39^, prompting their selection for this investigation. However, further exploration is needed to elucidate how habitat preference and diet may influence the bioindicator capabilities of juveniles. Different trace element patterns of juveniles of nase, barbel, and chub were hypothesized. It was also predicted that the trace element concentrations would be the highest in juveniles with benthic habitat preference and higher trophic levels due to higher exposure from their microhabitat and diet. Hence, diverse patterns of trace element accumulation in juveniles of three cyprinids with various habitat preferences and diets were analyzed to test these hypotheses. At the same time, the potential risks of fish consumption on human health were also evaluated. Moreover, the bioindicator potential of juveniles was evaluated to investigate the suitability of the studied species as indicators of trace element pollution specifically during the polluted period in 2013, which was selected as the focal year due to its heightened contamination levels.

## Results

### Biological features of fish

A total of 15 cyprinids were investigated. The mean standard length and standard deviation of nase, barbel, and chub were 72.3 ± 3.8 mm, 52.1 ± 2.4 mm, and 60.6 ± 5.1 mm, respectively. The mean body weight and standard deviation of nase, barbel, and chub were 5.57 ± 1.10 g, 2.56 ± 0.32 g, and 4.06 ± 1.23 g, respectively. According to the length and weight data, the studied fish were categorized as the 0 + age class (juveniles)^67,68^. The trophic level of nase, barbel, and chub were 2.0, 3.1, and 2.7, respectively^69^.

### Characterization of the study area

The descriptive statistics of the concentrations of trace elements in water, obtained from the database of the National Environmental Information System of Hungary (OKIR in Hungarian), are presented in Table 1. The mean concentrations of Cu, Pb, and Zn were above the criterion chronic concentrations (CCCs) for the freshwater of the National Recommended Water Quality Criteria prescribed by the USEPA^70^ (Table 1). The annual mean concentration of Cd remained below the threshold; however, the monthly mean concentrations surpassed the CCCs multiple times in 2013 (Table 1).

**Table 1:** Descriptive statistics of total trace element concentrations in water and sediment samples from the Szamos River obtained from the database of the National Environmental Information System of Hungary (OKIR in Hungarian) and Málnás et al.^66^ and Simon et al.^11^, respectively for the year 2013.

Element	Trace element concentration (μg l^−1^) in water			Threshold values (μg l^−1^)^a^
	Min.	Max.	Mean ± SD	
Cd	0.20	1.4*	0.58 ± 0.44	0.72
Cr	1	12	3.2 ± 3.7	74
Cu	5*	20*	9.5 ± 5.7*	3.1
Fe	45	658	366 ± 227	1000
Mn	1.8	388	146 ± 111	–
Pb	3*	20*	6.9 ± 6.3*	2.5
Zn	1	270*	136 ± 94*	120

Element	Trace element concentration (mg kg^−1^ dry weight) in sediment			Toxicity classification of sediment^b^
	Min.	Max.	Mean ± SD	
Cr	18	57	32 ± 21	moderately polluted
Cu	32	111	58 ± 40	heavily polluted
Fe	12,773	25,997	15,323 ± 6656	non-polluted
Mn	632	1961	1009 ± 667	heavily polluted
Pb	40	87	49 ± 24	non-polluted
Sr	11	35	17 ± 12	–
Zn	537	1073	646 ± 283	heavily polluted

For the water data, the average concentrations was used from monthly measurements taken between April and November 2013. Málnás et al.^66^ and Simon et al.^11^ collected and analysed the physical and chemical parameters of the sediment from the same sites and the same period than in the present study.

^a^Criterion chronic concentrations (CCCs) for the freshwater of National Recommended Water Quality Criteria^70^.

*Concentration of trace elements in the water was higher than the threshold value.

^b^According to the toxicity classification of sediments by Baudo and Muntau^71^.

The descriptive statistics of the concentrations of trace elements in the sediments obtained from previous studies^11,66^ are presented in Table 1. The mean concentration of Cr represented the sediments as moderately polluted. In contrast, the mean concentrations of Cu, Mn, and Zn classified the sediment status as heavily polluted, according to the toxicity classification of sediments^71^.

### Macroelements

In this study, the macroelements (Ca, K, Mg, Na) and trace elements (Cd, Cr, Cu, Fe, Mn, Pb, Sr, Zn) were separately evaluated due to their various physiological and bioaccumulation features. The mean concentrations of macroelements in muscle, gills, and liver are summarized in Table 2.

**Table 2:** Concentrations of trace elements in the muscle, gills, and liver (in mg kg^−1^ wet weight) of nase, barbel, and chub in the Szamos River (A) (mean value ± standard deviation).

(A) Trace element concentrations in different tissues				
Tissue	Element	Nase	Barbel	Chub
Muscle	Ca	354 ± 7.6^a^	337 ± 67^a^	377 ± 98^a^
	K	2517 ± 46^a^	1728 ± 299^b^	2495 ± 28^a^
	Mg	189 ± 11^a^	141 ± 17^b^	181 ± 17^a^
	Na	387 ± 31^a^	336 ± 58^a^	445 ± 33^b^
	Cd	BDL	BDL	0.10 ± 0.17
	Cr	0.12 ± 0.02^a^	0.18 ± 0.04^b^	0.12 ± 0.03^a^
	Cu	0.33 ± 0.08^a^	0.55 ± 0.07^b^	0.43 ± 0.08^a^
	Fe	7.4 ± 1.7^a^	11 ± 1.6^b^	9.1 ± 2.2^a,b^
	Mn	0.68 ± 0.06^a^	0.87 ± 0.13^a^	0.33 ± 0.08^b^
	Pb	0.16 ± 0.06^a^	0.19 ± 0.15^a^	0.36 ± 0.31^a^
	Sr	2.1 ± 0.22^a^	1.3 ± 0.22^b^	1.5 ± 0.31^b^
	Zn	6.3 ± 0.81^a^	11 ± 1.5^b^	14 ± 3.4^b^
Gills	Ca	3878 ± 178^a^	3059 ± 279^b^	6761 ± 3935^c^
	K	1401 ± 85^a^	1266 ± 737^a^	1395 ± 346^a^
	Mg	264 ± 21^a^	178 ± 52^b^	262 ± 50^a^
	Na	369 ± 24^a^	275 ± 83^a^	452 ± 166^a^
	Cd	BDL	BDL	0.07 ± 0.13
	Cr	0.39 ± 0.09^a^	0.53 ± 0.10^a^	0.26 ± 0.06^b^
	Cu	0.42 ± 0.08^a^	1.1 ± 0.54^b^	0.84 ± 0.12^b^
	Fe	72 ± 60^a^	109 ± 97^a^	39 ± 19^a^
	Mn	13 ± 0.47^a^	11 ± 3.5^a,b^	7.4 ± 2.2^b^
	Pb	0.60 ± 0.20^a^	0.68 ± 0.46^a^	0.53 ± 0.68^a^
	Sr	29 ± 5.2^a^	14 ± 4.1^b^	55 ± 40^a^
	Zn	19 ± 1.1^a^	39 ± 11^b^	58 ± 8.9^c^
Liver	Ca	611 ± 213^a^	1192 ± 1002^a^	682 ± 362^a^
	K	2196 ± 120^a^	1626 ± 1601^a,b^	842 ± 402^b^
	Mg	256 ± 121^a^	131 ± 93^a,b^	57 ± 24^b^
	Na	572 ± 63^a^	411 ± 141^a,b^	329 ± 13^b^
	Cd	BDL	0.24 ± 0.53^a^	0.29 ± 0.42^a^
	Cr	2.5 ± 1.4^a^	1.4 ± 0.57^a^	0.67 ± 0.15^b^
	Cu	14 ± 4.5^a^	3.9 ± 0.77^b^	2.2 ± 1.3^b^
	Fe	61 ± 14^a,b^	71 ± 18^a^	36 ± 17^b^
	Mn	3.1 ± 1.3^a^	5.2 ± 3.8^a^	1.8 ± 0.70^a^
	Pb	3.2 ± 1.2^a^	2.1 ± 2.0^a^	0.93 ± 0.52^b^
	Sr	1.7 ± 0.53^a^	1.3 ± 0.69^a^	1.4 ± 0.48^a^
	Zn	30 ± 11^a^	74 ± 55^a^	51 ± 27^a^

BDL: Below detection limit.

^a,b,c^The values with different letters in the same row are significantly different (Mann–Whitney U test, *p* < 0.05).

The Kruskal–Wallis test revealed significant differences among the three species concerning the concentrations of K, Mg, and Na (*p* < 0.05) in muscle, Ca and Mg (*p* < 0.05) in gills, and K, Mg, and Na (*p* < 0.05) in the liver (Table 2).

In muscle, the lowest concentrations of K and Mg for barbel, while the highest concentrations of Na for chub were recorded (*p* < 0.05). In gills, the highest concentration of Ca for chub and lowest concentrations of Mg for barbel were measured (*p* < 0.05). In the liver, the concentrations of K, Mg, and Na were significantly higher in nase compared to chub (*p* < 0.05) (Table 2).

There was a significant (*p* < 0.05) decrease in the concentrations of some macroelements with trophic levels (Table 3). K concentrations in muscle, Na concentrations in the liver, and Mg concentrations in muscle and gills were negatively correlated with trophic levels (Table 3).

**Table 3:** Correlation coefficients between trace element concentrations in tissues of species and trophic levels of species in the Szamos River, significant at *p* < 0.05 (N = 15).

Elements	Tissues		
	Muscle	Gills	Liver
K	− 0.794	n.s	n.s
Mg	− 0.794	− 0.567	n.s
Na	n.s	n.s	− 0.416
Cr	0.624	n.s	n.s
Cu	0.813	0.756	− 0.549
Fe	0.699	n.s	n.s
Sr	− 0.813	− 0.643	n.s
Zn	0.605	0.472	n.s

n.s.: Non-significant.

### Essential and non-essential trace elements

The mean concentrations of the trace elements in muscle, gills, and liver are presented in Table 2. The concentrations of Cd in the muscle, gills, and liver of nase and the muscle and gills of barbel were below the detection limit. In general, the highest concentrations of Cd, Cr, and Pb were measured in the liver, while Fe, Mn, and Sr reached the highest concentrations in the gills. The concentrations of Cu and Zn were highest in the liver of nase and barbel, respectively. However, Zn concentrations were most elevated in the gills of chub, and Cu concentrations in the gills of nase were lower than those of chub (Table 2). The trace element concentrations were generally lowest in the muscle in all the studied fish species (Table 2).

The Kruskal–Wallis test detected significant differences among the species in the trace element concentrations of Cd, Cr, Cu, Fe, Mn, Sr, and Zn (*p* < 0.05) in the muscle, Cd, Cr, Cu, Mn, Sr, and Zn (*p* < 0.05) in the gills, and Cd, Cr, Cu, Fe, and Pb (*p* < 0.05), in the liver.

In the case of muscle, the highest concentrations of Cr and Cu were found in barbel, the highest concentrations of Sr in nase, while concentrations of Cd were highest in chub (Mann–Whitney U test, *p* < 0.05) (Table 2). The lowest concentrations of Mn were found in chub, and Zn concentrations were the lowest in nase (Mann–Whitney U test, *p* < 0.05) (Table 2). In the case of gills, the highest concentrations of Cd and Zn were measured in chub (Mann–Whitney U test, *p* < 0.05) (Table 2). The lowest concentrations of Cr were found in chub, those of Cu and Zn were measured in nase, while the concentrations of Sr were lowest in barbel (Mann–Whitney U test, *p* < 0.05) (Table 2). In the case of the liver, the highest concentrations of Cu were found in nase (Mann–Whitney U test, *p* < 0.05) (Table 2). The lowest concentrations of Cd were measured in nase, while Cr and Pb accumulated in the lowest concentrations in chub (Mann–Whitney U test, *p* < 0.05) (Table 2).

Furthermore, the concentrations of several trace elements showed a significant increase in terms of the trophic level of fish (*p* < 0.05) (Table 3). The concentrations of Cr, Cu, Fe, and Zn in muscle and concentrations of Cu and Zn in gills showed a significant (*p* < 0.05) positive correlation with the trophic level (Table 3). The concentrations of Sr in muscle and gills and those of Cu in the liver showed a significant (*p* < 0.05) decrease with trophic levels (Table 3).

**Figure 1:**
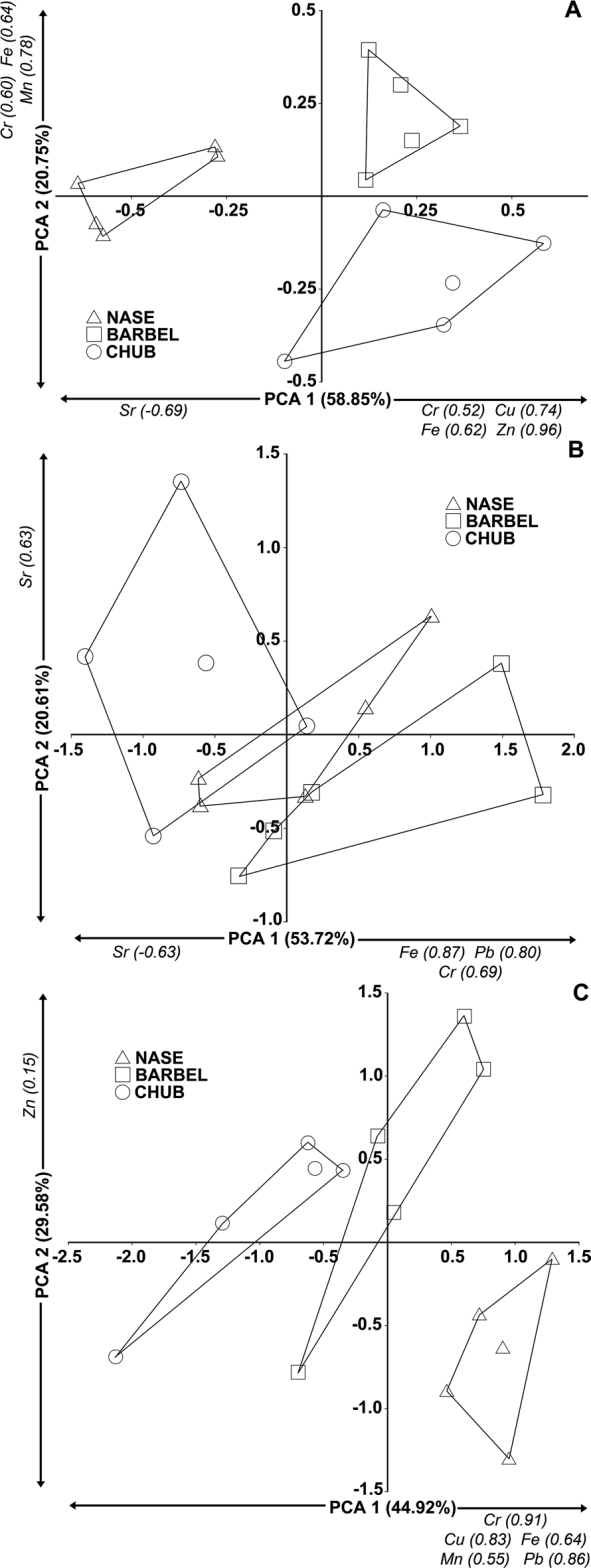
The principal component analysis of trace element concentrations (mg kg^−1^, wet weight) in the muscle (**A**), gills (**B**), and liver (**C**) of the three cyprinid species from the Szamos River. The elements exhibiting significant correlations with the PCA1 and PCA2 axes and their respective Spearman’s rho values are indicated.

The PCA showed clear separations among the different species based on trace element patterns in muscle (Fig. 1A) and the liver (Fig. 1C). In the case of muscle, the first component (PCA 1) contributed 58.85% of the total variance, while the second (PCA 2) contributed 20.75% of the total variance (Fig. 1A). In the case of liver, the first component contributed 44.92% of the total variance, the second (PCA 2) contributed 29.58% of the total variance (Fig. 1C). The trace element patterns in gills were also well separated among the different species. However, nase showed a minimal overlap with barbel and chub (Fig. 1B). In the case of gills, the first component (PCA 1) explained 53.72% of the total variance, while the second component (PCA 2) described the 20.61% of the total variance (Fig. 1B). The elements exhibiting significant correlations with the PCA1 and PCA2 axes, along with their respective Spearman's rho values, are indicated on Fig. 1.

### Bioconcentration factor and metal pollution index

The bioconcentration factors for water and sediments are summarized in Table 4. In general, the highest BCF values for water for Cd, Cr, Cu, and Pb were observed in the liver. At the same time, the highest BCF values for water for Fe and Mn were found in the gills (Mann–Whitney U test, *p* < 0.05).

**Table 4:** The bioconcentration factor (BCF) as the ratio of the mean concentration of trace elements (mg kg^−1^ wet weight) in the specific tissue to its mean concentration in the water (mg l^−1^) (A), and in the sediment (mg kg^−1^ dry weight) (B) (mean value ± standard deviation).

Elements	Tissue	Nase	Barbel	Chub
(A) Bioconcentration factors for water				
Cd	Muscle	–	–	168 ± 301
	Gills	–	–	126 ± 219
	Liver	–	415 ± 927^a^	511 ± 726^a^
Cr	Muscle	38 ± 6.7^a^	58 ± 12^b^	38 ± 9.3^a^
	Gills	122 ± 27^a^	166 ± 31^b^	81 ± 18^a^
	Liver	778 ± 454^a^	443 ± 179^a^	210 ± 47^b^
Cu	Muscle	35 ± 8.9^a^	59 ± 7.3^b^	46 ± 8.3^a^
	Gills	44 ± 8.9^a^	119 ± 57^b^	88 ± 13^b^
	Liver	1445 ± 473^a^	409 ± 80^b^	229 ± 134^b^
Fe	Muscle	20 ± 4.7^a^	31 ± 4.4^b^	25 ± 6.0^a,b^
	Gills	196 ± 165^a^	296 ± 265^a^	106 ± 51^a^
	Liver	166 ± 37^a,b^	193 ± 48^a^	98 ± 46^b^
Mn	Muscle	4.6 ± 0.38^a^	6.0 ± 0.87^a^	2.3 ± 0.52^b^
	Gills	90 ± 3.2^a^	75 ± 24^a,b^	51 ± 15^b^
	Liver	21 ± 8.8^a^	36 ± 26^a^	12 ± 4.8^a^
Pb	Muscle	23 ± 8.8^a^	28 ± 21^a^	51 ± 44^a^
	Gills	87 ± 29^a^	98 ± 67^a^	77 ± 99^a^
	Liver	458 ± 175^a^	306 ± 281^a,b^	134 ± 75^b^
Zn	Muscle	46 ± 6.0^a^	80 ± 11^b^	99 ± 25^b^
	Gills	139 ± 8.3^a^	284 ± 83^b^	425 ± 65^c^
	Liver	220 ± 83^a^	540 ± 403^a^	376 ± 197^a^
(B) Bioconcentration factors for sediment				
Cr	Muscle	0.37 ± 0.07^a^	0.57 ± 0.11^b^	0.38 ± 0.09^a^
	Gills	1.2 ± 0.27^a^	1.6 ± 0.31^a^	0.80 ± 0.18^b^
	Liver	7.7 ± 4.5^a^	4.4 ± 1.8^a^	2.1 ± 0.47^b^
Cu	Muscle	0.58 ± 0.15^a^	0.96 ± 0.12^b^	0.75 ± 0.14^a^
	Gills	0.73 ± 0.15^a^	2.0 ± 0.94^b^	1.5 ± 0.21^b^
	Liver	24 ± 7.8^a^	6.7 ± 1.3^b^	3.8 ± 2.2^b^
Fe	Muscle	0.05 ± 0.01^a^	0.07 ± 0.01^b^	0.06 ± 0.01^a,b^
	Gills	0.47 ± 0.39^a^	0.71 ± 0.63^a^	0.25 ± 0.12^a^
	Liver	0.40 ± 0.09^a,b^	0.46 ± 0.12^a^	0.23 ± 0.11^b^
Mn	Muscle	0.07 ± 0.01^a^	0.09 ± 0.01^a^	0.03 ± 0.01^b^
	Gills	1.3 ± 0.05^a^	1.1 ± 0.34^a,b^	0.74 ± 0.22^b^
	Liver	0.31 ± 0.13^a^	0.51 ± 0.38^a^	0.18 ± 0.07^a^
Pb	Muscle	0.32 ± 0.13^a^	0.40 ± 0.30^a^	0.73 ± 0.63^a^
	Gills	1.2 ± 0.41^a^	1.4 ± 0.95^a^	1.1 ± 1.4^a^
	Liver	6.5 ± 2.5^a^	4.4 ± 4.0^a,b^	1.9 ± 1.1^b^
Sr	Muscle	12 ± 1.3^a^	7.6 ± 1.3^a^	8.8 ± 1.8^a^
	Gills	169 ± 30^a^	83 ± 24^a^	315 ± 234^a^
	Liver	8.4 ± 3.1^a^	7.4 ± 4.0^a,b^	7.9 ± 2.8^b^
Zn	Muscle	0.98 ± 0.13^a^	1.7 ± 0.23^b^	2.1 ± 0.52^b^
	Gills	2.9 ± 0.17^a^	6.0 ± 1.8^b^	9.0 ± 1.4^c^
	Liver	4.7 ± 1.8^a^	11 ± 8.5^a^	7.9 ± 4.2^a^

For the water data, the average concentrations was used from monthly measurements taken between April and November 2013. Málnás et al^66^ and Simon et al.^11^ collected and analysed the physical and chemical parameters of the sediment from the same sites and the same period than in the present study.

^a,b,c^The values with different letters in the same row are significantly different (Mann–Whitney U test, *p* < 0.05).

In the case of muscle, the highest BCF values for water for Cd were found in chub, while those for Cr and Cu were measured in barbel. The lowest BCF values for water for Mn were in chub and those for Zn were found in nase (Mann–Whitney U test, *p* < 0.05). In gills, the highest BCF values for water for Cd and Zn were in chub, while those for Cr were in barbel. At the same time, the lowest BCF values for water for Cu and Zn were in nase (Mann–Whitney U test, *p* < 0.05). In the liver, the highest BCF values for water for Cu were in Nase, while the lowest for Cd were found in nase, and those for Cr were in chub (Mann–Whitney U test, *p* < 0.05) (Table 4A).

Similar tendencies were also observed in the BCF values for the sediments (Mann–Whitney U test, *p* < 0.05) (Table 4B). In addition, the BCF values for the sediments for Sr were highest generally in the gills (Mann–Whitney U test, *p* < 0.05).

**Figure 2:**
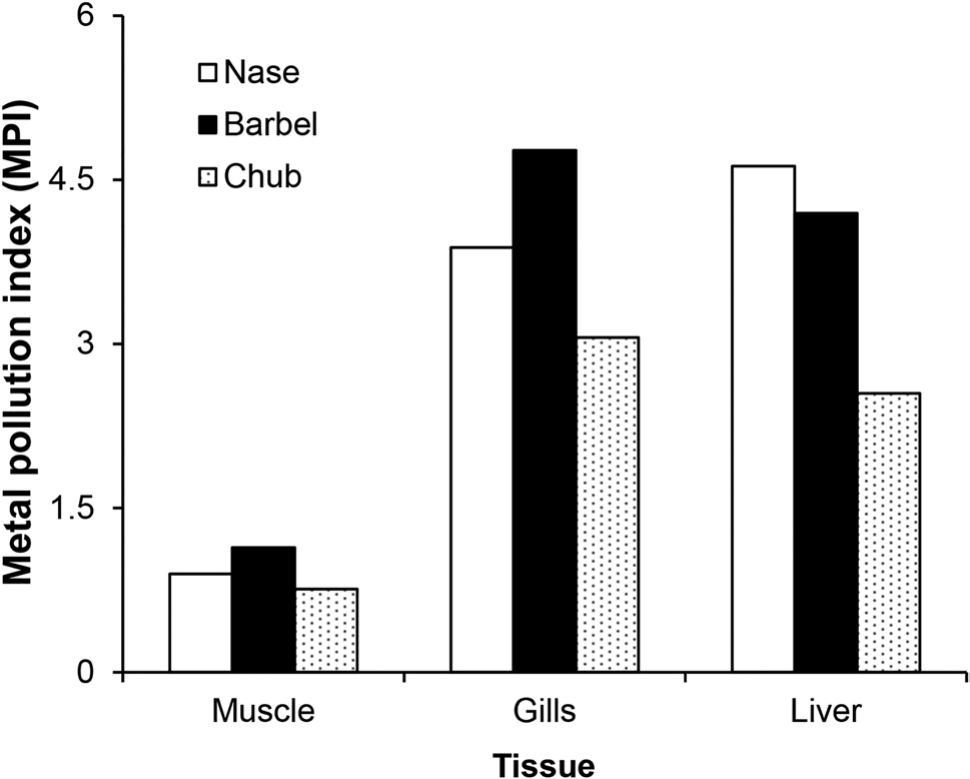
Metal pollution index (MPI) in the different organs of cyprinid juveniles from the Szamos River.

The metal pollution index (MPI) values of muscle and gills were highest in barbel, while the MPI of the liver was highest in nase (Mann–Whitney U test, *p* < 0.05) (Fig. 2.). The lowest MPI values were observed in chub in all examined organs (Mann–Whitney U test, *p* < 0.05) (Fig. 2.).

### Human health implications

The mean concentrations of Cd and Pb in the chub muscle were over the EU’s limit: Cd (0.05 mg kg^−1^ ww) and Pb (0.30 mg kg^−1^ ww)^72^. Additionally, concentrations of the other analyzed trace elements in the muscle of the studied species were found to be below the Maximum Allowable Concentrations (MACs in mg kg^−1^ ww): Cd (0.05), Pb (0.30)^72^, Cr (1.0), Cu (30.0), Fe (43.0), Mn (1.0), and Zn (40.0)^73^.

## Discussion

Both our study and earlier research have emphasized that the Szamos River experienced substantial trace element pollution in 2013, originating from industrial, mining, and agricultural activities^11,18,66^. The trace element pattern of sediments is a stable indicator of long-term pollution of rivers, while that of water reflects recent pollution^74^. The sediments of the Szamos River were moderately polluted with Cr and heavily polluted with Cu, Mn, and Zn, indicating that this aquatic ecosystem has been continuously polluted by these elements^11,66^. At the same time, the concentrations of Cu, Cd, Pb, and Zn in the river’s water exceeded the CCCs for the freshwater of EPA^70^ in 2013. According to the water chemistry data of the National Environmental Information System of Hungary (OKIR in Hungarian), the concentrations of these elements were approximately 2–10 times higher than those in previous years.

The distribution of macroelements (Ca, K, Mg, Na) in fish were found according to their biological functions. The highest concentrations of Ca and Mg were found in the gills because these elements play an essential role in forming and maintaining bony structures (i.e., arches and rakers in gills)^75^. Elevated concentrations of Na were measured in the liver of nase and barbel, while similarly high concentrations were detected in the gills of chub. This phenomenon can be attributed to the essential role of Na, an electrolyte, in liver functioning. Additionally, the gills serve as the primary site for Na uptake from water. ^18,76^. Similar results were observed in field studies^25,60,77^ and laboratory experiments with juveniles of common carp (*Cyprinus carpio* Linnaeus, 1758)^13^.

Among the investigated species, the herbivorous nase had the lowest trophic level, followed by the omnivorous chub, while the benthivorous barbel had the highest trophic level. The Cr, Cu, Fe, and Zn concentrations in muscle and Cu and Zn concentrations in gills increased with the trophic level. These results partially confirmed our first hypothesis that the concentration of trace elements would be highest in species with higher trophic levels. A central part of the positive correlation between trace element concentration and the trophic level was observed in the case of muscle, independently from their pollution status in water and sediment because the concentrations of Cu and Zn were over the limit of polluted levels in the water and sediment too^70,71^. At the same time, the Fe concentrations did not exceed these limits, and Cr was detected only in levels describing a moderately polluted status in the sediments^70,71^. According to these results, many trace elements in the muscle increased with trophic level, which indicated the significant influence of diet on the trace element patterns of the muscle of juveniles^18,36,38^. During the last decade, several authors also highlighted the importance of the muscle tissue in bioaccumulation and biomagnification of trace elements in the case of juveniles^18,29,34,38,39^.

In addition, some trace elements did not show a correlation between their concentrations and the trophic level, despite significant differences, especially in the gills and liver. Moreover, recent studies also revealed that habitat preference has a more substantial influence on the trace element pattern of juveniles than the trophic level itself^33,78,79^. This phenomenon may further support our results since the concentrations of the central part of trace elements did not differ between the benthic, herbivorous nase, and benthivorous barbel (except for Cu). In contrast, they differed between these benthic species and the pelagic, omnivorous chub. Previous studies conducted in Serbian water bodies, including the Danube River, Kačer River, Zaovine, and Medjuvršje reservoirs, also proved elevated Cu concentrations in the liver of nase^22,35,60^. Nase feeds exclusively on benthic algae, and according to published data, high amounts of Cu can accumulate in green algae and diatoms^80,81^. The significant negative correlation between Cu concentrations in the liver and trophic level among the investigated cyprinids can potentially be attributed to the specific diet of nase. This is because, among the species studied, nase exhibits the lowest trophic level.

The non-essential (or toxic) trace elements negatively affect health even at low concentrations, e.g., Cd and Pb^82^. Cd was detected in elevated concentrations in the tissues of pelagic chub, while its concentrations were below the detection limit in the gills of nase and barbel and the liver of nase. The Cd concentration of the water of the Szamos River was approximately ten times higher in 2013 than in previous years. It exceeded the CCCs during several months in 2013 based on the water chemistry data from the National Environmental Information System of Hungary (OKIR in Hungarian). The elevated concentration of Cd in water was reflected in the trace element pattern of pelagic chub, whereas its impact on benthic juveniles was less pronounced. Similar patterns were observed in multispecies investigations, wherein elevated Cd concentrations in the water primarily accumulated in pelagic species^52,79^. At the same time, Pb concentrations were highest in the liver of benthic juveniles. In general, habitat preference accounts for the trace element pattern of gills^6,18^. However, both Cd and Pb concentrations were elevated in the water, but the primary source of Pb accumulation in fish is the food consumed, while fish take up Cd directly from the water through their gills^29,83^. According to this phenomenon, toxic trace elements that primarily enter fish through their gills from water have a more significant effect on the trace element pattern of pelagic species than on that of benthic species^6,24,52^. These results confirmed our second hypothesis that habitat preference influences trace element patterns.

Several mechanisms make fish juveniles effective bioindicators of trace element pollution of aquatic ecosystems, e.g., relative higher growth and metabolic rate, relative food intake and quantity of respiratory water passing through the gills, and inadequately developed detoxification system^18,29,38,78^. The primary objective of this study was to examine the bioindicator potential of various juveniles and evaluate the influence of their diet and habitat preferences on it within a polluted aquatic ecosystem. Cu and Zn polluted both the water and the sediment. In this case, when the microhabitats of species were relatively equally contaminated, the diet significantly affected the bioaccumulation of these elements. However, in the case of pelagic chub, the Zn concentrations in the gills were higher than in the liver. The highest Zn concentrations can generally be found in the liver^35,84,85^. This revealed that relatively higher Zn concentrations in the gills of a pelagic species could reflect a recent Zn pollution^15,86,87^. Cr and Mn were in moderately and heavily polluted concentrations in the sediments, respectively, while their concentrations did not exceed the CCCs in the water. These elements bioaccumulated in higher concentrations in the benthic species (nase, barbel) than in the pelagic chub. Cd concentrations stayed over the CCCs of water for several months, while Cd did not accumulate in the sediments. As a result of this phenomenon, Cd was found at higher concentrations in tissues of the pelagic chub than in benthic nase and barbel.

These results highlighted that habitat preference influenced the bioindicator capability of juveniles more than the diet^29,78^. However, the diet and trophic level of nase, barbel, and chub differed significantly, but in larvae and early juvenile stages, they fed on the same prey^40^. Fish larvae onsets the exogenous feeding after the depletion of yolk reserves^41^. Most larvae and early juveniles feed exclusively on zooplankton, showing an ontogenic shift in feeding later^45^. Juveniles commence feeding on their species-specific prey items several months after hatching^45^. Therefore, in juveniles, due to the similar feeding habits during the early stages of the lifespan, the diet may not affect the trace element patterns as much as in the case of adults^18,35,36^. However, the investigated juveniles were caught from the same section of the Szamos River; different environmental trace element exposure impacted them. Because fish with benthic habitat preference frequently cause bioturbation, which can increase trace element release from the sediment^88^. Consequently, the trace element pattern of the microenvironment of a benthic and a pelagic fish is different, even in the same water body. Trace elements could also interact with each other during their uptake by fish through biological barriers, such as the gills or digestive system. Furthermore, habitat preference developed earlier than the final species-specific diet in the case of juveniles. Based on the results, we consider that the habitat preference of juveniles can seriously impact the bioaccumulation of trace elements. According to the phenomena mentioned above, the bioaccumulation patterns of trace elements in juveniles with various habitat preferences can be utilized as effective bioindicators of trace element pollution of aquatic ecosystems. Thus, the simultaneous use of pelagic and benthic juveniles can be very helpful also in applied monitoring programs for determining water and sediment pollution status.

In the case of muscle, the mean concentrations of Cd and Pb in the chub were over the prescribed MACs. Interestingly, the concentrations of these toxic elements were also over the CCCs in the water, while their concentrations were not in a polluted status in the sediments. This also followed our finding that the pelagic chub was more sensitive to recent water pollution than the benthic nase and barbel. Hungary has a size limit for both investigated cyprinids, and anglers can harvest only adults. Nevertheless, higher concentrations of Cd and Pb in the muscle of chub juveniles can be dangerous to the ecosystem (due to biomagnification in the food web) and human health (due to illegal poaching). Therefore, further detailed examinations of trace element patterns of adult fish from the Szamos River are required to assess human health risks.

## Conclusions

This study provides new information on the ecotoxicology of water, sediments, and juveniles of nase, barbel, and chub from the Szamos River. Overall, the trace element concentrations observed in both water and sediments were notably elevated, with some exceeding threshold concentration values. This highlights the significant pollution burden of the Szamos River in 2013. The accumulated trace element patterns of the juveniles showed significant differentiation. The various habitat preferences and diets could explain these differences. However, only a few trace element concentrations showed correlations with the trophic levels, especially in the case of muscle. At the same time, several differences were found among the species related to habitat preference. The pelagic chub had a higher affinity for bioaccumulation of trace elements, which exceeded the threshold concentrations of water. In contrast, the benthic nase and barbel had a higher affinity for trace elements that exceeded the sediment threshold concentrations. These may reflect different exposure of the juveniles with various habitat preferences due to their different microhabitat and several mechanisms, e.g., bioturbation. As a result of recent trace element pollution of water, the concentrations of toxic Cd and Pb in the muscle tissue of pelagic chub exceeded the prescribed MACs by the EU. Therefore, it is advisable to monitor the health risks associated with the consumption of fish living in the Szamos River, particularly by sampling adult individuals. Nase, barbel, and chub have an important role in the food web, angling, and commercial fishing, in addition to their widespread status in the rivers of Europe. According to our results, future monitoring studies should simultaneously focus on juveniles or adults with various habitat preferences as adequate bioindicators of trace element pollution of water and sediments. However, further studies are needed to identify such patterns in other habitats.

## Material and methods

### Ethical approval

The Workplace Animal Experiments Committee of Debrecen University approved the experimental protocol and the end-points of the experiments. All methods were carried out following relevant national and international guidelines and regulations (permission number: HBH/01/00971-2/2013). The study complies with the Animal Research: Reporting of in Vivo Experiments (ARRIVE) guidelines.

### Study area and sample collection

The Szamos River is one of the largest tributaries of the Tisza River. Its watershed collects water over a 15,882 km^2^ area of Romania and Hungary. Its basin is strongly affected by anthropogenic activities, such as mining and industrial emissions, and thus, it is continuously polluted by trace elements^11,18^. Fish were collected at the settlement, Csenger, near the Hungarian-Romanian border. The geocoordinates of the sampling site are N47.838303 and E22.693744.

For this study, a total of 15 juveniles of nase, barbel, and chub (5 individuals per fish species) were collected in November 2013 by electrofishing (Hans Grassl IG200/2b, PDC, 75–100 Hz, 350–650 V, max. 10 kW, Hans Grassl GmbH, Germany). The sample size for each group was designed similarly to the study by^35^. Additionally, the fisheries society authorized the collection of up to 5 specimens per species. The fish were transported in containers with aerated river water to the laboratory. The standard length (SL) and the total weight (W) of each specimen were measured to the nearest 0.1 mm and 0.01 g, respectively. After the measurements, the fish were sacrificed immediately by spinal severance and stored at -18 °C until sample processing.

### Sample processing and element analysis

Muscle samples were dissected from the dorsolateral muscle of the left side. The second gill arch from the left side was collected as a gill sample. After the incision of the abdominal wall, the whole liver was sampled. During the dissection, sterile plastic tools were used to avoid any trace element contamination. The dissected samples were rinsed with double deionized water (Milli-Q), and their wet weight (WW) was measured into glass beakers using an analytical balance (Precisa 240A, Switzerland). The samples were dried overnight at 105 °C. The samples were afterward digested on an electric hot plate using 4.0 ml 65% (m/m) nitric acid (reagent grade, Merck, USA) and 1.0 ml 30% (m/m) hydrogen-peroxide (reagent grade, Merck, USA) in the same container at 80 °C for 4 h. The digested samples were then diluted with 1% (m/m) nitric acid (reagent grade, Merck, USA) and Milli-Q water up to a final volume of 10 ml^89,90^.

The Ca, K, Mg, Na, Cd, Cr, Cu, Fe, Mn, Pb, Sr, and Zn concentrations were measured by a microwave plasma-atomic emission spectrometer (MP-AES 4200, Agilent Technologies, USA) system. The values of the limits of detection (LOD) in μg l^−1^ were: Ca, 0.070; K, 0.270; Mg, 0.080; Na, 0.484; Cd, 1.863; Cr, 0.489; Cu, 0.414; Fe, 0.856; Mn 0.057; Pb, 1.235; Sr, 0.051; and Zn, 3.125, respectively. Each sample’s detection limit was verified based on the instrumental detection limit, sample mass, and volume to which it had been diluted. The average detection limits for each of the assessed elements were (mg kg^−1^ wet weight): Ca, < 0.001; K, 0.002; Mg, 0.001; Na, 0.004; Cd, 0.009; Cr, 0.004; Cu, 0.003; Fe, 0.006; Mn < 0.001; Pb, 0.009; Sr, < 0.001; and Zn, 0.023, respectively. The following wavelength lines of the MP-AES analysis were used: Ca 422.673 nm, K 766.491 nm, Mg 285.213 nm, Na 588.995 nm, Cd 228.802 nm, Cr 425.433 nm, Cu 324.754 nm, Fe 371.993 nm, Mn 403.076 nm, Pb 405.781 nm, Sr 407.771 nm, and Zn 481.053 nm.

We applied a five-point calibration procedure prepared from the multi-element standard solution (Merck ICP multi-element standard solution IV). An autosampler (Agilent SPS4, USA), a Meinhard-type nebulizer, and a double-pass spray chamber were used. The measurement used a certified reference material (ERM-BB422, fish muscle, LGC Standards, UK). The recoveries were within 10% of the certified values for the metals. The wavelengths and measuring parameters were chosen based on suggestions provided by the instrument’s software (MP Expert). The concentrations of all elements were expressed as mg kg^−1^ wet weight (ww).

### Bioconcentration factors and metal pollution index

The bioconcentration factor (BCF) of a trace element is defined as the proportion of the concentration of that trace element in an organism (or in a specific tissue of the organism) to the concentration of the trace element in the water or the sediment^91^:

$${\text{BCF}} = {\text{C}}_{{{\text{fish}}}} /{\text{C}}_{{{\text{water}}}}$$

$${\text{BCF}} = {\text{C}}_{{{\text{fish}}}} /{\text{C}}_{{{\text{sediment}}}}$$

where C_fish_ means the concentration of the trace element in the whole body or tissue of the organism, expressed as mg kg^−1^ wet weight, C_water_ is the concentration of the trace element in the water, described as mg l^−1^., and C_sediment_ is the concentration of the trace element in the sediment, expressed as mg kg^−1^ dry weight.

The trace element concentration data for water were sourced from the database of the National Environmental Information System of Hungary (OKIR in Hungarian). For this study, we utilized the average concentrations derived from monthly measurements conducted in 2013. Specifically, data from April to November were used because of this period is important regarding the lifespan of the studied juveniles. The trace element concentrations of sediment from the Szamos River from 2013 were used based on earlier studies ^11,66^. References^11,66^ collected and analyzed the physical and chemical parameters from the same sites and the same period than in the present study. The water data were compared with criterion chronic concentrations (CCCs) for the freshwater of the National Recommended Water Quality Criteria^70^. The trace element concentrations in the sediments were evaluated according to the toxicity classification of sediments by Baudo and Muntau (2020).

Metal pollution index (MPI) was assessed to compare the total content of trace elements (Cd, Cr, Cu, Fe, Mn, Pb, Sr, Zn), excluding the macroelements for the different tissues of the different species. The MPI formula is the following^92,93^:

$${\text{MPI}} = \left( {{\text{C}}_{{1}} \times {\text{C}}_{{2}} \times {\text{C}}_{{3}} \times \ldots {\text{C}}_{{\text{n}}} } \right)^{{{1}/{\text{n}}}} ,$$

where C_n_ is the mean concentration of trace element *n* in the analyzed tissue (mg kg^−1^ wet weight).

### Statistical analysis

The data were presented as mean values accompanied by standard deviation. The statistical analysis was performed with IBM SPSS Statistics for Windows (Version 20.0)^94^ and Past 4.0^95^. Before analyses, the normal distribution and homogeneity of variances were tested with Shapiro–Wilk and Levene’s tests, respectively. The non-parametric Kruskal–Wallis test was used to evaluate the differences in the trace element concentrations and BCF values of trace elements in the tissues of nase, barbel, and chub. As a post hoc test, a Mann–Whitney U test was used to explore the significant differences. Spearman’s rank correlation test was used to study the relationship between element concentrations and trophic levels of fish in order to explore possible biomagnification features of elements in juveniles^96,97^. The trophic levels were obtained from the database of FishBase^69^. A principal component analysis (PCA) was used to assess the differentiation of species based on the concentration of trace elements (Cd, Cr, Cu, Fe, Mn, Pb, Sr, Zn) of the studied tissues, except for the macroelements. Spearman’s rank correlation test was conducted between the concentration of trace elements and the component scores of the first two PC axes to explore variables significantly affecting the differentiation of species.

To assess the risk for human consumption of nase, barbel, and chub from the Szamos River, trace element concentrations of muscle (filet) were compared with the maximum acceptable concentrations (MACs) established by the European Union^72^ and the Food and Agriculture Organization of the United Nations^73^.

## Data Availability

The datasets used and/or analysed during the current study available from the corresponding author on reasonable request.
